# Sleep Quality and Influencing Factors of Nurses in Fever Clinics During Closed-Loop Management: An Exploratory Mixed-Methods Study

**DOI:** 10.3390/healthcare14111507

**Published:** 2026-05-29

**Authors:** Fenglin Wang, Yue Hu, Dongli Wei, Fengqin Zhou, Yilan Liu, Weixian Wang

**Affiliations:** 1 Department of Infection, Union Hospital, Tongji Medical College, Huazhong University of Science and Technology, 1277 Jiefang Dadao, Wuhan 430022, China; wangfenglin@whuh.com (F.W.); weidongli@whuh.com (D.W.); wangweixian@whuh.com (W.W.); 2 Medical Services, Union Hospital, Tongji Medical College, Huazhong University of Science and Technology, Wuhan 430022, China; 2022xh0084@hust.edu.cn; 3 Department of Nursing, Union Hospital, Tongji Medical College, Huazhong University of Science and Technology, Wuhan 430022, China; 2004xh0900@hust.edu.cn

**Keywords:** frontline healthcare workers, occupational stress, psychological capital, sleep quality, COVID-19, mixed-methods research

## Abstract

**Highlights:**

**What are the main findings?**

- Nurses working under closed-loop COVID-19 management experienced moderate sleep disturbances (PSQI score: 8.16 ± 4.25).
- Sleep quality was significantly associated with night shifts, work-related stress, and psychological capital (self-efficacy, resilience, hope, and optimism).

**What are the implications of the main findings?**

- Targeted psychological support and stress management strategies may be beneficial for improving sleep quality among frontline nurses.
- Humanized and holistic management approaches may enhance mental well-being and healthcare workforce sustainability during public health crises.

**Abstract:**

Background: During the COVID-19 pandemic, fever clinic nurses under closed-loop management faced high occupational stress and strict isolation, which may impair sleep quality. However, evidence in this population remains limited. This study investigated the sleep quality of fever clinic nurses during closed-loop COVID-19 management and identified associated factors. Methods: A sequential explanatory mixed-methods design approach was employed. Quantitative data were collected using the Pittsburgh Sleep Quality Index (PSQI) and Nurses’ Psychological Capital Scale from 33 front-line nurses, acknowledging the limited sample size. Semi-structured in-depth interviews were conducted with six nurses to provide qualitative insights. Results: The mean PSQI score was 8.16 ± 4.25, indicating moderate sleep disturbances among nurses. Factors associated with sleep quality included demographic and occupational factors (e.g., night shifts, work pressure) and psychological capital (self-efficacy, resilience, hope, and optimism). Qualitative analysis identified three themes: the impact of personal circumstances on sleep quality, psychological pressures during closed-loop management, and the role of self-regulation in coping. Conclusions: During the closed-loop management for COVID-19 pandemic prevention and control, the sleep quality of nurses in fever clinics was poor. This study identified personal circumstances, work pressure, and psychological capital as potential factors associated with sleep quality, suggesting that further research is needed to develop and test targeted interventions. These findings provide preliminary evidence that may inform future management strategies, but do not support definitive intervention recommendations.

## 1. Introduction

Nurses played a critical role in patient care and infection control during the COVID-19 pandemic. Fever clinic nurses were assigned to perform screening and treatment under isolation and protective conditions during the epidemic and therefore were both physically and psychologically challenged. This placed them at risk for chronic sleep deprivation [1,2]. A meta-analysis reported that 43% of frontline nurses experienced sleep disturbances [3]. Sleep is a form of rest that anyone needs, with the development of sleep disturbances directly affecting sleep quality. Poor sleep quality not only reduces immunity but also impairs cognitive function and emotional stability, threatening both nurse well-being and patient safety. Studies have shown that sleep deprivation in nurses seriously threatens their physical and mental health and the safety of patient care, and may even lead to work fatigue and resignation [4,5,6].

It is well established that many factors have an influence on sleep. According to a Chinese study, a greater number of night shifts worked by nurses is associated with worse sleep quality [7]. Another study revealed that the sleep quality of nurses correlated positively with anxiety and depression [8]. As surveyed by Wang et al. [1], clinical front-line nurses treating COVID-19 patients had poor sleep, with sleep quality being associated with anxiety, stress load, and lower self-efficacy in completing work. An Italian study also examined the effect of shift work on the sleep patterns of clinical nurses [9], and concluded that their gender, work stress, and level of social support affected sleep quality. In addition, a Turkish study reported that during the COVID-19 epidemic, 50.4% of medical staff were found to have symptoms of insomnia, which had a considerable impact on their psychological and physical health [10]. Psychological capital is a positive state and represents a combination of four psychological states: self-efficacy, hope, optimism, and resilience [11]. These factors exert a positive effect on nurses’ psychology, emotion, health, and work attitude and competence. There is also evidence that residence, outdoor activity, and gender are risk factors for sleep quality [12].

During the normalized management of the pandemic, closed-loop management was implemented for fever clinic staff to unify management and minimize infection risk, involving strict restrictions on activities and living arrangements. The concept of closed-loop management was developed by Kaplan and Norton in 2008 [13], who divided closed-loop management into five stages: strategy formulation, transformation, and implementation, supervised learning, and strategy inspection and adjustment. During the COVID-19 pandemic, staff in the isolation area were managed in a closed-loop manner to unify management [14,15]. However, most existing studies on nurse sleep during the pandemic have focused on frontline nurses in general. The specific sleep quality and influencing factors for nurses subjected to the unique and stringent conditions of closed-loop management—particularly those in fever clinics, which served as the critical first point of contact—remain underexplored.

Fever clinic nurses were the key personnel subjected to closed-loop management during the COVID-19 pandemic. During this period, nurses were under work and body pressures, but lacked emotional support and ways to relieve negative emotions. This aggravated their anxiety, depression, and other negative emotions, all of which affected their sleep quality [8]. In addition, nurses were confined to a very limited number of activities during closed-loop management, resulting in their psychological pressure being substantial during this period. A Turkish study [16] reported that a high level of social and family support was a protective factor for good sleep quality, while traumatic stress increased under a low level of social support, leading to poorer sleep quality. Yang et al. [17] considered that when an individual was faced with pressure, was confident of making a success, had the will and approach to solve problems, had a realistic and flexible attitude, and was able to recover quickly from adversity all correlated negatively with sleep disturbances. Psychological capital reflects an individual’s self-efficacy, hope, optimism, and perseverance in the future and focuses on the individual’s self-management capabilities in the face of future adversity, thereby promoting personal growth. In order to alleviate psychological anxiety and pressure during closed-loop management for preventing and controlling the pandemic, an individual needs to possess sufficient psychological resources. The aims of the current study were to investigate the sleep status of nurses in fever clinics under the specific condition of closed-loop management, and to analyse its relationship with factors such as psychological capital. This would provide a reference for the management of fever clinics during this unique and high-pressure operational mode.

Therefore, this study aimed to answer the following research question: What is the sleep quality of nurses in fever clinics during closed-loop management, and what are the key influencing factors, including psychological capital, work-related factors, and personal circumstances?

## 2. Materials and Methods

### 2.1. Survey Participants and Methods

The study combined quantitative and qualitative research methods (sequential explanatory mixed-methods design). Quantitative investigation and analyses were carried out initially to identify sleep quality and influencing factors, and then a qualitative research scheme was designed based on the quantitative results to provide in-depth explanations and supplement the findings. Quantitative research provides the basis for sample selection and developing a semi-structured interview design for qualitative research, with the qualitative research results then used to explain and supplement the results of the quantitative research [18]. This study adopted an sequential explanatory mixed-methods design grounded in a pragmatic paradigm. The qualitative component was informed by a descriptive phenomenological orientation to better understand nurses’ lived experiences during closed-loop management. Accordingly, Colaizzi’s phenomenological analysis method was used for interview data analysis.

### 2.2. Quantitative Research

#### 2.2.1. Research Design, Participants, and Data Collection

Using a convenience sampling method, 33 supporting nurses working in the fever clinic of a Grade 3 first-class hospital (the highest level in the Chinese hospital tier system, providing comprehensive medical services, teaching, and research) in Wuhan from February to June 2022 were selected for the questionnaire survey. Inclusion criterion included supporting nurses who had worked in the fever clinic for more than one month, while the exclusion criterion included those who failed to complete the questionnaire or provided an invalid questionnaire. The sample size was limited by the number of nurses under closed-loop management in the fever clinic during the study period; all eligible nurses were invited to participate to ensure maximum representation. For the qualitative interviews, six nurses were selected from the 33 survey respondents using purposive sampling to ensure variation in demographic and occupational characteristics, including age, marital status, years of working experience, and sleep quality levels. Participants were selected to provide diverse perspectives regarding sleep experiences during closed-loop management. Interview recruitment continued until thematic saturation was reached and no substantially new concepts emerged.

#### 2.2.2. Questionnaire Survey Procedures

An electronic questionnaire was generated through Wenjuanxing (Changsha Ranxing Information Technology Co., Ltd., Changsha, China; https://www.wjx.cn/, accessed on 17 May 2026), with the QR code of the questionnaire sent to the nurses under closed-loop management of the fever clinic. A unified guideline was adopted to explain the purpose of the research and its contents, the method used to fill in the questionnaire, and the principle of anonymity. Before the formal survey was started, the author learned how to score the Pittsburgh Sleep Quality Index (PSQI) scale and the Nurses’ Psychological Capital Scale as well as how to analyse the test results and also conduct a pre-survey on one respondent. After confirming that the contents of the questionnaire and the data collected were appropriate and accurate, the final questionnaire was sent to the remaining respondents. Part I of the questionnaire contained the informed consent form, and elaborated on the content and purpose of the research and the requirements for filling in the scales. The respondents answered the questions after carefully reading the informed consent form and deciding whether or not to participate in the survey. To improve the quality of completing the questionnaire, the following requirements needed to be met: (1) each IP address could only submit the questionnaire once to avoid duplicate responses; (2) the questionnaire needed to be filled in completely with the time taken being longer than five minutes before submission; (3) if the answers to all questions were the same, the questionnaire was considered to be invalid.

#### 2.2.3. Outcomes and Measures

The primary dependent variable was sleep quality, measured using the Chinese version of the Pittsburgh Sleep Quality Index (PSQI). Both the overall PSQI score and its sub-dimensions were analyzed, with the overall PSQI score considered the primary outcome. Psychological capital, as a potential predictor, was measured by the Nurses’ Psychological Capital Scale. Specifically, PSQI, compiled by the psychiatrist Buysse from the University of Pittsburgh, was used mainly for assessing the sleep quality of the respondents over the past month, and included seven dimensions: subjective sleep quality (6), sleep latency (2, 5a), sleep duration (4), habitual sleep efficiency (1, 3, 4), sleep disturbance (5b–5j), use of sleep medication (7), and daytime dysfunction (8, 9). Each dimension was assigned with a score from 0 to 3 based on its severity, with the cumulative score of each respondent for all seven dimensions calculated as the total score of the PSQI scale (0–21 points). A total score greater than 7 points suggested problems with sleep quality. Higher score indicate worse sleep quality. Previous studies reported that the total Cronbach’s α coefficient and split-half reliability coefficient for the Chinese version of the PSQI were both 0.87 [19]. In the current study sample, the Cronbach’s α coefficient for the PSQI was 0.68, indicating acceptable internal consistency.

#### 2.2.4. Possible Predictors

The independent variables included socio-demographic characteristics, occupational factors, and psychological capital dimensions. Socio-demographic variables included age, gender, marital status, and having children. Occupational variables included working experience, night shifts, perceived work pressure, days in closed-loop management, and vaccination status. Psychological factors included self-efficacy, hope, resilience, and optimism measured using the Nurses’ Psychological Capital Scale. We also investigated the following variables, which reflected the characteristics of the participants’ occupational environment and individual ability to handle COVID-19 [20], years of working experience, living in Wuhan (yes or no), clinical experience on infection diseases (yes or no), days in closed-loop management, times working in the night during closed-loop management, felt pressure during daily work (yes or no), and whether the participants had received three doses of COVID-19 vaccines. In addition, we investigated the following factors which reflected the participant’s subjective or objective ability to cope with sleep difficulties [21,22]; frequency of physical activity at least 30 min per week, and individual psychological capital measured by Lathan’ psychological capital scale.

The Nurses’ Psychological Capital Scale was developed by Luthans et al. with its Chinese translation revised by He Zhonghua in 2010, which has since been used widely by nurses [23]. The scale consisted of four dimensions and 20 items: self-efficacy (items 1–6), hope (items 7–12), resilience (items 13–17), and optimism (items 18–20). A six-point Likert scale was used as the scoring method for the questionnaire, ranging from “strongly disagree (1 point)” to “strongly agree (6 points)”. Higher sums of the scores indicated better psychological capital. Previous studies reported a Cronbach’s α coefficient of 0.914 for the Chinese version of the scale, with all four subscales demonstrating coefficients above 0.80 [23]. In the current study sample, the Cronbach’s α coefficient for the Nurses’ Psychological Capital Scale was 0.95, indicating excellent internal consistency. Reliability coefficients for all subscales are presented in Supplementary Table S1.

#### 2.2.5. Conceptual Framework

**Figure 1:**
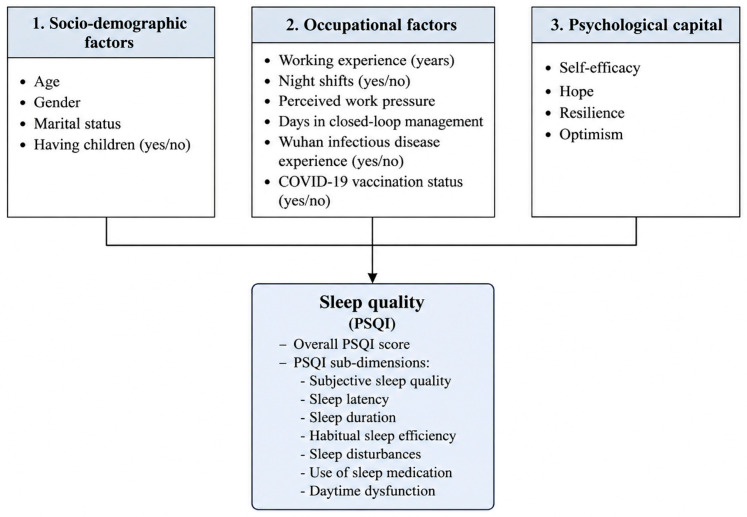
Conceptual framework of factors potentially associated with sleep quality among nurses during closed-loop management.

Based on previous literature and the study objectives, we developed an analytical framework to examine factors potentially associated with sleep quality among nurses during closed-loop management. The framework categorized independent variables into socio-demographic characteristics, occupational stressors, and psychological resources, with sleep quality measured by the PSQI considered the primary dependent variable (Figure 1). Occupational stressors and psychological capital dimensions were selected based on prior evidence suggesting their associations with sleep disturbances among frontline healthcare workers during the COVID-19 pandemic.

#### 2.2.6. Statistical Analysis

The analytical framework of this study examined associations between socio-demographic, occupational, and psychological factors and sleep quality among nurses during closed-loop management. For the descriptive statistics, categorical variables were expressed as numbers (percentage) and continuous variables as mean ± standard deviation (SD). To identify factors associated with sleep quality, we conducted a series of linear regression analyses with PSQI scores or subdimension scores as the dependent variables. The full linear regression models included all the candidate predictors listed above. That these analyses are exploratory and should be interpreted cautiously given the ordinal nature of these variables and the limited sample size. A stepwise approach based on Akaike information criterion (AIC) was then used to select variables included in the final regression models. AIC is a statistical method used to compare different possible models and determine which one was the best fit for the data [24]. Given the exploratory nature of this study and the relatively large number of candidate predictors compared with the sample size, this procedure was used to reduce model complexity and identify the most informative variables. However, we acknowledge that stepwise regression has known limitations, including potential overfitting and unstable variable selection; and therefore the findings should be interpreted as exploratory and hypothesis-generating rather than confirmatory. All candidate predictors considered in the initial regression models were selected based on theoretical relevance and prior literature, including sociodemographic variables (e.g., age, marital status), occupational factors (e.g., night shifts, work pressure), and psychological factors (e.g., self-efficacy, hope, resilience, and optimism). Given space constraints, detailed stepwise selection processes are not presented; however, all candidate predictors are described above, and the final selected variables are reported in Section 3. The stepwise regression on multiple PSQI subscales may increases the risk of identifying chance findings; therefore, the subscale results should be considered exploratory. No formal multiple-testing adjustment was applied because the analyses were considered exploratory and hypothesis-generating rather than confirmatory. Therefore, the findings, particularly for PSQI subdimension analyses, should be interpreted cautiously. All analyses were performed using R, version 3.6.0. Two-sided *p*-values and 95% confidence intervals (CIs) were reported throughout. *p* < 0.05 was considered statistically significant. Although PSQI component scores are ordinal variables with limited ranges, linear regression was used as an exploratory analytical approach because these scores have commonly been treated as approximately continuous in prior sleep-related research. Given the small sample size, more complex ordinal regression models were considered unlikely to provide stable estimates. Model assumptions for linear regression were evaluated prior to interpretation. Multicollinearity was assessed using variance inflation factors (VIFs) for the final regression models retained after stepwise AIC selection, and no evidence of problematic multicollinearity was observed. Residual plots and Q–Q plots were visually inspected to assess homoscedasticity, linearity, and normality assumptions.

### 2.3. Qualitative Research

#### 2.3.1. Interviewed Subjects

Interview subjects were selected from the above 33 respondents using the purposive sampling method. A total of six nurses were interviewed. Thematic saturation was considered achieved when no new themes or insights emerged from the analysis of consecutive interviews, and the data collected provided sufficient depth and redundancy to understand the core experiences related to sleep quality and influencing factors.

#### 2.3.2. Interview Method

Based on the literature review, group discussion, and consultation with experts, an outline of the interview was initially drawn up, with the final outline determined after a pre-interview with a nurse. The contents are listed below: (1) How did you feel when working in the fever clinic during closed-loop management? (2) Did you sleep well during closed-loop management? (3) During the closed-loop period, what factors do you think affected your sleep? (4) What difficulties and pressures did you encounter whilst working in the fever clinic and what measures did you take to deal with them? Due to the closed-loop management, the interview was conducted as a one-on-one video call with the consent of the interviewee. Data were collected via a semi-structured interview, with the whole process recorded. The interview lasted for 15–20 min. To protect their privacy, the names of the interviewees were replaced by numbers.

#### 2.3.3. Data Analysis Method

Within 24 h after the end of the interview, the recordings were converted into text data for encoding and classification. Colaizzi’s seven-step analysis method was used for organizing and analysing the data and refining the interview topics, as detailed below [25]. Valuable statements in the text were identified by the researcher through repeated and careful reading of the text and word-for-word analysis; recurring viewpoints were encoded; encoded viewpoints were brought together to find common features/similarities and then clustered into prototype themes; the themes were linked with the actual situation of the research object for a detailed and appropriate description; each prototype theme was defined and described with some typical original statements extracted from or inserted into the description of each prototype theme; similar prototype themes and their descriptions were compared repeatedly to identify and extract similar viewpoints and construct themes; the sorted text data and extracted themes were provided to the interviewees to verify their authenticity and integrity, and the missing data were supplemented based on feedback from the respondents. Coder roles: Two researchers (the lead author and a co-author with expertise in qualitative research) independently coded the interview transcripts. Discrepancies were resolved through discussion and consensus with a third researcher. Triangulation: Data triangulation was achieved by comparing qualitative findings with quantitative results from the PSQI and psychological capital scales, as well as by using multiple data sources (interviews and survey data). Trustworthiness: In addition to member checking, we conducted peer debriefing sessions with two experienced qualitative researchers not involved in the study to review themes and interpretations. An audit trail of coding decisions and theme development was maintained to enhance dependability and confirmability.

#### 2.3.4. Researcher Reflexivity

Prior to and during the interview process, the research team engaged in reflective practices to acknowledge and bracket potential biases. The primary interviewer was a nurse manager with experience in clinical settings but not directly involved in the management of the fever clinic participants, aiming to reduce social desirability bias. The team recognized that their own experiences during the COVID-19 pandemic could influence data interpretation. To mitigate this, during data analysis, team members consistently questioned their assumptions and returned to the raw interview transcripts to ensure themes were grounded in the participants’ narratives rather than the researchers’ preconceptions. This reflexive stance was maintained to enhance the trustworthiness of the qualitative findings.

## 3. Results

### 3.1. Sleep Quality of Nurses in the Fever Clinic During Closed-Loop Management

The results of the study showed (Table 1) that the total PSQI score of 8.16 (4.25) during the closed-loop management period of fever clinic nursing staff indicated that poor sleep quality was observed of fever clinic during the closed-loop period, and that the personal circumstances such as age, gender, marital status, whether they were from Wuhan, number of days of the closed-loop period, whether they were working night shifts, and whether they had completed the three doses of the vaccine, were associated with sleep quality of the nursing staff of the fever clinic. In addition, we found that self-efficacy, resilience, hope, and optimism of nurses were associated with good sleep quality (Table 2 and Table S2). Due to the very small number of male nurses (*n* = 2, 6.2%) in the sample, the observed associations related to gender (e.g., female gender associated with lower use of sleep medication and less daytime dysfunction) should be interpreted with extreme caution. These findings are not statistically stable and are not generalizable due to the severe sample imbalance.

### 3.2. Interview Results

The qualitative findings were used to further explain and contextualize the quantitative results regarding sleep quality and its associated factors. Table 3 summarizes the major themes, subthemes, and representative quotations derived from the qualitative interviews. Three major themes emerged: the influence of personal circumstances on sleep quality, psychological pressure during closed-loop management, and the role of self-regulation during closed-loop management.

Some respondents considered that personal circumstances had a certain impact on their sleep. Nurse A stated “I worked for six hours in the fever clinic. During my off time, if I had nothing to do during the day and couldn’t leave the hotel, I would sleep. However, sleeping too much during the day affected my sleep at night”. Nurse D reported “I was during the first few days of closed-loop management, but after a week, I became a little restless and felt like I had lost my freedom”. Nurse E said “In my regular ward, I worked one night shift per week. In the fever clinic, I worked two night shifts per week, and later three. I believe this impacted my sleep”. Nurse F reported “My child is just over a year old. I missed him very much. Throughout the closed-loop management, I couldn’t not take care of him. He didn’t even recognize me during our video calls (sadly)”. These findings supported the quantitative results showing that night shifts, marital status, and living circumstances were associated with PSQI scores and sleep-related outcomes.

Most of the respondents believed that during closed-loop management, changes in work style and living environment caused some psychological pressure. Nurse B confessed “Working in the fever clinic was stressful for a period, especially when there were confirmed cases. I was a somewhat worried about getting infected. The fever clinic is a special frontline post. There was considerable pressure when intubating or rescuing a patient with high fever or a suspected case.”. Nurse C reported “I was afraid of encountering critically ill patients or special situations I hadn’t experienced before. Sometimes, patients had a bad attitude, which also caused me some pressure”. Nurse D reported “Similar to the emergency department, the fever clinic didn’t receive patient information in advance. A patient in serious condition might arrive suddenly by car, and many unforeseen situations awaited us.”. Nurse F stated “I still felt somewhat worried working the night shift alone in the cabin. If a critically ill patient arrived, I felt a bit powerless. I don’t think this night shift arrangement was particularly conducive to peace of mind.”. This theme further explained the quantitative association between perceived work pressure and poorer sleep-related outcomes, particularly daytime dysfunction and sleep disturbances.

The qualitative narratives further indicated that adaptive self-regulation strategies, such as exercise, emotional adjustment, and maintaining structured daily activities, may have functioned as psychological coping mechanisms that buffered stress-related sleep impairment. This finding highlights the potential importance of behavioral and emotional resilience in maintaining sleep quality during prolonged crisis management. For example, Nurse A said “In the hotel, I could do some exercise. If the intensity was high, I slept better”. Nurse C reported “I can adjust my emotions relatively quickly; nothing bothers me for too long. During my first few days in the fever clinic, I didn’t sleep well at night and woke up easily. Later, I adapted to the new environment and could sleep well”. Nurse E stated “Since I had to take the supervisor exam this year, I would finish the day shift at two o’clock, return to the hotel for a nap, and then study. Having study tasks made it manageable”. Nurse F confessed “As nurses, night shifts are part of our job. We have to accept it through self-regulation; someone always has to cover the night shift. Working night shifts definitely affects sleep. On the day after a night shift, don’t schedule any other activities—just rest well”. Overall, the qualitative findings contextualized and enriched the quantitative results by illustrating how occupational stressors, personal circumstances, and psychological capital influenced nurses’ sleep experiences during closed-loop management. These narratives complemented the quantitative findings that higher psychological capital, particularly resilience and hope, was associated with better sleep quality and adaptive coping.

## 4. Discussion

This study provides novel evidence on sleep quality and its influencing factors among fever clinic nurses under closed-loop management, a context characterized by unique occupational stressors and isolation measures. By integrating quantitative associations with qualitative narratives, this study provides a more comprehensive understanding of how occupational stressors, personal circumstances, and psychological capital jointly shaped nurses’ sleep experiences during closed-loop management. The qualitative findings not only contextualized the statistical associations observed in the regression analyses but also illustrated the lived experiences underlying sleep disturbances, including anxiety related to night shifts, emergency uncertainty, and emotional isolation. The qualitative findings expanded the quantitative results by illustrating the lived experiences underlying the observed statistical associations. For example, while quantitative analyses identified night shifts and work pressure as factors associated with poorer sleep quality, the interviews revealed that emergency uncertainty, fear of infection, isolation, and disrupted daily rhythms contributed substantially to psychological strain and sleep disturbances. Similarly, the qualitative narratives demonstrated how self-regulation, emotional adjustment, and adaptive coping reflected the protective role of psychological capital observed in the regression analyses. The study showed that the total PSQI score of the fever clinic nurses during closed-loop management was 8.16 ± 4.25, indicating that they had poor sleep quality during this period. This finding aligns with the survey results on the sleep quality of frontline nurses during epidemic prevention and control reported by Zhuang et al. [5] and can be interpreted through the lens of the conservation of resources theory, which suggests that prolonged exposure to stressors (e.g., isolation, high workload) depletes personal resources, leading to sleep disturbances. Our results showed that personal circumstances such as age, gender, marital status, living in Wuhan, the number of closed-loop days, working night shifts, and receiving three-shot vaccination were factors that influenced the sleep quality of nurses in fever clinics. These factors reflect the interplay between individual vulnerability and environmental demands, consistent with the job demands–resources model. We also found that several dimensions of psychological capital were significantly associated with sleep quality and specific sleep-related domains, although the direction and strength of these associations varied across subscales. This finding is consistent with those of a longitudinal study on positive psychological capital and sleep at the University of Hong Kong [26], and supports the broaden-and-build theory, which posits that positive psychological resources enhance coping and well-being, thereby protecting sleep quality. Positive psychological resources may buffer the emotional exhaustion caused by prolonged uncertainty, social isolation, and high-intensity clinical work. Nurses with greater resilience and hope may be better able to cognitively reframe stressful experiences and maintain emotional regulation, thereby reducing hyperarousal and facilitating sleep recovery under prolonged occupational stress. Given the multiple regression models performed on PSQI total score and subdimensions, we acknowledge the increased risk of false-positive findings. Therefore, the results, particularly those for subscale analyses, should be interpreted as exploratory and hypothesis-generating rather than confirmatory.

The sleep status of fever clinic nurses during closed-loop management may be related to the special nature of their work, such as strict restrictions on activities of the nurses, patients in critical conditions, and the possibility of potential infection. In this study, the use of sleep medication was reported by a small number of participants, and the linear regression analysis indicated that age was significantly associated with the use of sleep medication. This finding suggests that older nurses may be more likely to use sleep medication, potentially due to the complex work and living environment, as suggested by Zhao [27], though this finding should be interpreted with caution due to the small sample size and the limited statistical power of the analysis. Male nurses in our sample were associated with better sleep quality in some sub-dimensions, but this finding is based on a very small number of male participants (*n* = 2) and should not be considered representative or generalizable; it is presented here solely as a preliminary observation for potential hypothesis generation in future research with balanced samples. In this study, marital status showed a statistically significant association with sleep quality in the regression model, but this finding should be interpreted cautiously given the sample size and the potential for unmeasured confounding. Due to the particularity of nursing work, it is inevitable for nurses to have workday and night shifts, with night shifts directly disrupting their sleep rhythms. Under closed-loop management, the impact of night shifts may have been amplified because nurses were unable to restore normal circadian routines through family interaction, outdoor activities, or environmental changes after work. The combination of circadian disruption, prolonged confinement, and psychological vigilance may therefore have intensified sleep disturbances beyond those observed in conventional shift-work settings. In this study, poor sleep quality in fever clinic nurses during the closed-loop period were associated with night shifts, and Chen et al. [7] reported similar findings. Our study also found associations between sleep quality and whether nurses lived in Wuhan or had completed a three-dose COVID-19 vaccination series, but these associations may reflect complex social and contextual factors and require confirmation in larger, more diverse samples. This trend may have been related to Wuhan’s rich experience in dealing with the pandemic and the protective effect of the vaccine. Fever clinics are outposts for screening patients with COVID-19, where patients experience complex conditions and mostly require emergency rescues, which results in greater challenges and pressures to nursing work. The higher the occupational stress load perceived by individuals, the higher the incidence of sleep disturbances and worse sleep quality. Importantly, the qualitative findings suggested that the stress experienced by nurses was not limited to workload itself, but also involved unpredictability, fear of sudden emergencies, infection-related anxiety, and perceived responsibility during isolated night shifts. These stressors may contribute to sustained cognitive and physiological hyperarousal, which is a recognized mechanism underlying insomnia and impaired sleep quality. However, there is evidence that the positive psychological capital of individuals and its dimensions correlate positively with the coping style they take under stress [28]. These findings suggest an association between psychological capital and sleep quality, indicating that future research could explore whether targeted psychological support strategies might enhance psychological capital and thereby improve sleep quality. However, this remains a hypothesis that requires further intervention-based studies to confirm.

According to the quantitative and qualitative analyses, the nurses considered that the main factors affecting their sleep at night were the rotation of night shifts and the psychological pressure of facing emergencies. These qualitative insights provided contextual explanations for the quantitative associations observed in the regression analyses and clarified how occupational stressors translated into impaired sleep experiences during closed-loop management. This qualitative finding aligns with our quantitative results, which showed that the number of night shifts was significantly associated with the use of sleep medication (coefficient: −0.02, 95% CI: −0.04 to −0.01, *p* = 0.009) and that work pressure was significantly associated with daytime dysfunction (coefficient: 1.75, 95% CI: 0.56 to 2.94, *p* = 0.006), indicating objective links between night shift frequency, perceived stress, and sleep quality. As indicated in a previous study [29], night shift rotation reduces sleep, leading to insomnia or excessive sleepiness, as well as a reduced ability to adapt to the environment. There were many emergencies in fever clinics, which led to higher requirements for the emergency response ability and psychological quality of nurses. Better emergency response ability and psychological quality would result in less pressure during the emergency, with the quality of sleep correlating significantly with the level of pressure [16]. Regarding the manifestations of poor sleep quality during the closed-loop management, nurses reported difficulties in falling asleep and maintaining sleep, and also short sleep duration. This is consistent with our quantitative findings, where the mean scores for sleep latency (1.59 ± 1.19) and sleep duration (1.12 ± 1.39) indicated moderate to high levels of disturbance. At least 76% of the 233 nurses surveyed in Thailand reported the same conditions [30], although that survey was limited to nurses in various clinical departments of the hospitals. In our study, the nurses surveyed were in the same environment and faced the same stressful events, although the effects on them were different, as reflected in the variability of PSQI scores (SD: 4.25) across participants.

Finally, by integrating quantitative associations with qualitative experiences, this study suggests that sleep quality among fever clinic nurses during closed-loop management was shaped not only by measurable occupational and psychological factors, but also by subjective experiences of stress, isolation, uncertainty, and adaptive coping. Rather than focusing solely on workload reduction, future interventions may need to address the broader psychosocial environment of closed-loop management, including emotional isolation, perceived uncertainty, and opportunities for psychological recovery after night shifts. These findings highlight the potential value of supportive organizational environments and psychologically informed management strategies for maintaining nurse well-being during prolonged public health emergencies. Potential organizational strategies could include optimizing night-shift scheduling, providing designated psychological support or peer-support programs during closed-loop management, and ensuring adequate opportunities for rest and recovery after prolonged high-intensity work periods. Structured stress-management interventions and flexible recovery arrangements may also help mitigate the psychological burden associated with prolonged isolation and emergency care responsibilities. However, what has not been determined is how and to what extent each of these factors affected sleep. In subsequent studies, corresponding interventions could be taken according to the influencing factors to improve the sleep quality of nurses during closed-loop management.

A limitation of this study was that the nurses were surveyed over the same period and came from the same hospital, with differences in the management systems of various hospitals potentially resulting in different results. In addition, there were sporadic cases in Wuhan during the investigation period, which also created a certain pressure on medical institutions and medical staff that may have affected the results. Because female participants predominated in this study, it was not possible to analyze gender effects; therefore, caution should be exercised when generalizing the findings to male nurses. The small sample size (*n* = 33) limited the statistical power to detect significant associations and increased the risk of overfitting in the regression analyses, especially given the number of predictors examined. The use of convenience sampling introduced selection bias, as participants were not randomly selected from the target population. Furthermore, the study was conducted in a single hospital in Wuhan, which restricts the external validity and generalizability of the findings to other settings, regions, or healthcare systems. Therefore, results should be interpreted with caution and validated in larger, multi-center studies with more rigorous sampling methods.

## 5. Conclusions

During the closed-loop management for COVID-19 pandemic prevention and control, the sleep quality of nurses in the studied fever clinic appeared to be compromised. These exploratory findings suggest that more attention could be paid to the mental health of nurses in such settings. The establishment of a humanized management system and the provision of humanistic care might be beneficial strategies to explore for improving nurse well-being and sleep quality during closed-loop management. Further research with larger, multi-center samples is needed to confirm these observations and elucidate the causal relationships among the identified factors.

## Figures and Tables

**Table 1:** Basic description of the quantitative data.

Variable	Mean ± SD or Number (%)
Age (years)	30.78 ± 6.79
Gender (female)	30 (93.8%)
Working experience (years)	8.88 ± 7.73
Marital status (married)	16 (50%)
Having children (yes)	13 (40.6%)
From Wuhan (yes)	22 (68.8%)
Clinical experience with infectious diseases (yes)	5 (15.6%)
Days in closed-loop (days)	48.09 ± 21.00
Number of night shifts worked	10.91 ± 12.04
Weekly frequency of physical activity (≥30 min)	1.62 ± 1.50
Have received three doses of COVID-19 vaccines (yes)	23 (71.9%)
Feel pressures during daily work (yes)	26 (81.2%)
Psychological capital scores	78.78 ± 19.59
Self-efficacy	24.53 ± 6.77
Hope	23.69 ± 5.74
Resilience	19.28 ± 5.37
Optimism	11.28 ± 3.37
PSQI	8.16 ± 4.25
Subjective sleep quality	1.31 ± 0.78
Sleep latency	1.59 ± 1.19
Sleep duration	1.12 ± 1.39
Habitual sleep efficiency	0.5 ± 0.84
Sleep disturbances	1.06 ± 0.62
Use of sleep medication	0.22 ± 0.61
Daytime dysfunction	2.34 ± 1.47

SD: Standard deviation; PSQI: Pittsburgh sleep quality index.

**Table 2:** Multivariable linear regression models for PSQI total score and subdimensions.

Variable	Daytime dysfunction β (95% CI), *p*	Overall PSQI β (95% CI), *p*
Age (years)	0.06 (0.01, 0.10), 0.011	–
Gender (female)	–2.98 (−4.87, −1.10), 0.003	–
From Wuhan (yes)	–	0.85 (−1.81, 0.12), 0.083
Clinical infection experience (yes)	1.38 (0.14, 2.61), 0.030	–
Work pressure (yes)	1.75 (0.56, 2.94), 0.006	–
Resilience	−0.10 (−0.15, −0.05), 0.001	−0.22 (−0.38, −0.05), 0.012
Optimism	0.36 (0.10, 0.62), 0.008	–

β values represent unstandardized regression coefficients. Each column represents a separate final multivariable linear regression model obtained using AIC-based stepwise selection. Only variables retained in the final models are shown. Given the small sample size and multiple models, the results should be interpreted as exploratory and hypothesis-generating.

**Table 3:** Major qualitative themes, representative quotations, and integration with quantitative findings.

Theme	Description	Representative Quotation	Link to Quantitative Findings
Influence of personal circumstances on sleep quality	Nurses described how night shifts, family separation, restricted movement, and prolonged hotel isolation affected emotional well-being and disrupted sleep routines during closed-loop management.	“In the fever clinic, I worked two night shifts per week, and later three. I believe this impacted my sleep.” (Nurse E)	These findings supported the quantitative results showing that night shifts and personal circumstances were associated with PSQI scores and sleep-related outcomes.
Psychological pressure during closed-loop management	Participants reported stress related to emergencies, uncertainty, critically ill patients, infection risk, and insufficient psychological security during isolated night shifts.	“I still felt somewhat worried working the night shift alone in the cabin. If a critically ill patient arrived, I felt a bit powerless.” (Nurse F)	This theme further explained the quantitative association between perceived work pressure and poorer sleep-related outcomes, particularly daytime dysfunction.
Role of self-regulation and psychological capital	Nurses described coping strategies such as emotional adjustment, exercise, acceptance of night shifts, and maintaining study routines to adapt to stress and preserve sleep quality.	“I can adjust my emotions relatively quickly; nothing bothers me for too long.” (Nurse C)	These narratives complemented the quantitative findings that higher psychological capital, particularly resilience and self-efficacy, was associated with better sleep quality.

## Data Availability

As the data used in this study involve nurse-related information, they are subject to privacy and ethical restrictions. Therefore, the data are not publicly available. De-identified data can be provided upon reasonable request from the corresponding author, subject to approval by the institutional ethics committee.

## Supplementary Materials

The following supporting information can be downloaded at: https://www.mdpi.com/article/10.3390/healthcare14111507/s1, Table S1: Reliability analysis of the study instruments in the current sample; Table S2: Multivariable linear regression models for subdimensions.
