# Supplementary material for: Sleep Quality and Influencing Factors of Nurses in Fever Clinics During Closed-Loop Management: An Exploratory Mixed-Methods Study
Source: Healthcare (Basel). 2026 May 29;14(11):1507. doi: 10.3390/healthcare14111507 (PMC13256985; doi:10.3390/healthcare14111507)
Supplement: Supplementary file 1 [file healthcare-14-01507-s001.zip › healthcare-4247007-supplementary.pdf]

Supplementary Table S1. Reliability analysis of the study instruments in the current sample.

**Instrument**	**Dimension**	**Number of items**	**Cronbach's α**
PSQI	Total scale	7	0.68
Psychological Capital Scale	Total scale	20	0.95
	Self-efficacy	6	0.91
	Hope	6	0.84
	Resilience	5	0.84
	Optimism	3	0.74

PSQI: Pittsburgh Sleep Quality Index. Cronbach's α values ≥0.70 were considered acceptable indicators of internal consistency.

Supplementary Table S2. Multivariable linear regression models for subdimensions.

Variable	Subjective sleep quality β (95% CI), p	Sleep duration β (95% CI), p	Sleep efficiency β (95% CI), p	Sleep disturbances β (95% CI), p
Age (years)	–	−0.14 (−0.33, 0.05), 0.129	–	–
Gender (female)	–	−2.19 (−5.16, 0.78), 0.140	–	−1.05 (−1.85, −0.26), 0.012
Marital status (married)	0.55 (0.01, 1.10), 0.044	−1.49 (−3.92, 0.95), 0.218	–	–
From Wuhan (yes)	−0.67 (−1.27, −0.07), 0.029	1.37 (−0.03, 2.77), 0.055	−0.73 (−1.30, −0.16), 0.014	–
Days in closed-loop	–	−0.04 (−0.07, 0.00), 0.036	–	–
Night shift frequency	–	–	–	−0.02 (−0.04, −0.01), 0.009
COVID-19 vaccination (3 doses)	0.50 (−0.10, 1.10), 0.099	−1.82 (−3.25, −0.38), 0.016	0.57 (0.00, 1.14), 0.049	–

Work pressure (yes)	0.52 (−0.19, 1.22), 0.143	−1.13 (−2.81, 0.55), 0.175	0.67 (0.02, 1.32), 0.043	–
Self-efficacy	–	0.11 (−0.09, 0.32), 0.265	0.09 (0.03, 0.16), 0.007	–
Hope	–	–	0.11 (0.02, 0.20), 0.021	–
Resilience	–	–	−0.13 (−0.24, −0.02), 0.018	0.03 (−0.01, 0.07), 0.126
Optimism	0.10 (−0.02, 0.23), 0.111	−0.19 (−0.49, 0.11), 0.208	−0.16 (−0.31, 0.00), 0.053	0.18 (0.10, 0.26), <0.001

β values represent unstandardized regression coefficients. Each column represents a separate final multivariable linear regression model obtained using AIC-based stepwise selection. Only variables retained in the final models are shown. Given the small sample size and multiple models, the results should be interpreted as exploratory and hypothesis-generating.
